# Multitone Electrocochleography in Cochlear Implant Recipients: An Expert and Algorithm-Based Comparison

**DOI:** 10.1097/MAO.0000000000005043

**Published:** 2026-09-07

**Authors:** Hannes S. Hefty, Klaus Schuerch, Martin Kompis, Marco Caversaccio, Stefan Weder

**Affiliations:** a Department of Otorhinolaryngology, Head and Neck Surgery, Bern University Hospital; b Hearing Research Laboratory, ARTORG Center for Biomedical Engineering Research, Bern, Switzerland

**Keywords:** Binning algorithm, Cochlear implants, Expert analysis, Multitone electrocochleography, Objective analysis, Postoperative electrocochleography, Pure-tone electrocochleography

## Abstract

**Objectives:**

This study compared postoperative pure-tone and multitone electrocochleography (ECochG) recordings in cochlear implant recipients regarding signal characteristics and detectability of frequency-specific responses. The reliability of expert visual assessment and a Fourier transform–based binning algorithm were evaluated for detecting ECochG responses and identifying spectral components in multitone recordings.

**Study design:**

Prospective pilot cohort study.

**Setting:**

Tertiary referral hospital.

**Patients:**

Twenty-one cochlear implant recipients with stable residual hearing at least 6 months postimplantation.

**Interventions:**

Intracochlear cochlear microphonics potentials were recorded through the implant electrode array using pure tones at 250, 500, 1000, and 2000 Hz and 2 multitone stimuli comprising 3 or all 4 of the aforementioned frequencies. Measurements were conducted at least 6 months postimplantation, with follow-ups at 2 to 48 hours and 2 to 4 months thereafter.

**Main outcome measures:**

Response classification by the expert and the algorithm was compared. Inter-rater agreement, signal-to-noise ratio, sensitivity, and specificity were assessed.

**Results:**

Pure-tone ECochG responses were detected at all tested frequencies in every participant. Multitone stimulation produced an overall ECochG response in 17 of 21 participants. Response amplitudes and spatial patterns varied with electrode position and residual hearing. While tonotopic trends were observed, maximal responses were frequently shifted toward the basal cochlea. Inter-rater agreement was high for pure tones but clearly lower for frequency-specific components of multitone recordings, particularly at low frequencies. Signal-to-noise ratios were low across all conditions. The binning algorithm showed high sensitivity but limited specificity for pure tones and a frequency-dependent trade-off for multitone stimulation.

**Conclusions:**

In patients with stable audiometry and electrode position after at least 6 months of cochlear implant insertion, multitone ECochG was feasible and produced detectable responses. However, signal characteristics varied with electrode position and residual hearing, yielding more complex patterns than pure tones. This complexity hindered reliable identification of individual spectral components by experts and by objective methods.

In recent years, an increasing number of cochlear implant (CI) candidates have presented with substantial residual hearing before surgery. Preservation of this acoustic component is crucial because it supports improved speech perception, which represents one of the primary goals of CI treatment^1,2^.

In addition to delivering electrical stimulation, the CI electrode can also serve as a recording interface^3,4^. During CI implantation, it enables the direct acquisition of intracochlear potentials through electrocochleography (ECochG)^5,6^. In this context, ECochG has become an established method for monitoring inner-ear function both intraoperatively and postoperatively^7–9^.

Intraoperatively, a 500 Hz pure tone is commonly used as a surrogate marker for residual hearing^10–12^. Analyses typically focus on the cochlear microphonic (CM) amplitude^12,13^. A sudden reduction or loss of this signal is associated with poorer hearing preservation outcomes^14,15^. However, reliance on a single-frequency stimulus has inherent limitations. A response at 500 Hz does not reflect cochlear function across its full tonotopic extent^16^. Because this frequency corresponds to a part of the apical region, its sensitivity for detecting trauma in the basal turn is limited^3^. Moreover, not every reduction in CM amplitude indicates cochlear trauma^17^. For instance, decreases may occur when the electrode advances beyond the region of the characteristic frequency^18^. As the strongest response arises at the characteristic frequency, increasing the distance between the electrode and this region leads to physiological attenuation, which may result in misinterpretation^17^.

Postoperatively, ECochG also provides objective information, with recorded responses correlating with residual hearing and behavioral outcomes^7,19,20^. Nevertheless, obtaining a comprehensive cochlear profile requires multiple pure-tone measurements, rendering the procedure time-consuming.

A further challenge in both intraoperative and postoperative contexts is the reliance on expert visual inspection for signal interpretation^21^. Although objective analysis approaches for pure-tone ECochG responses exist, they have not achieved widespread clinical adoption^22^. Subjective interpretation introduces interobserver variability and limits comparability between implant centers.

To address these limitations, multitone stimulation has been investigated as an alternative approach. By simultaneously presenting multiple frequencies, multitone ECochG aims to provide a faster and more comprehensive representation of cochlear residual function, eliciting responses from several tonotopically distinct regions along the cochlea. Intraoperatively, multitone ECochG has been applied to detect cochlear trauma during electrode insertion^23,24^, with analyses typically based on fast Fourier transform (FFT)-derived amplitude changes^18^. Despite these developments, the clinical role of multitone ECochG remains insufficiently defined, and interpretation of the resulting complex recordings poses substantial challenges. Visual inspection, in particular, may introduce additional bias and reduce reproducibility, an issue that is amplified by the increased signal complexity inherent to multitone recordings^25^.

Against this background, the present study pursued 2 objectives: (1) to compare ECochG signals elicited by pure-tone and multitone stimulation, and (2) to evaluate the reliability of multitone ECochG interpretation by contrasting expert visual inspection with objective analysis methods. This was performed in a postoperative setting with minimal confounding factors, such as electrode movement and intraoperative cochlear trauma, in order to establish a clear interpretation framework before extending the research to the intraoperative setting.

## Methods

This prospective cohort study was conducted in a tertiary care hospital and adhered to the ethical standards outlined in the Declaration of Helsinki. Written informed consent was obtained from all participants, as approved by the local ethics committee (KEK-BE 2019-01578).

Twenty-one participants who had been implanted with a MED-EL device for at least 6 months, at which point residual hearing was assumed to have stabilized, were included. On the day of electrophysiological testing, pure-tone audiometry was performed in a certified acoustic chamber using a clinical audiometer (Interacoustics, Denmark). A low-frequency pure tone average (lfPTA) was calculated by averaging the thresholds at 125, 250, 500, and 750 Hz.

ECochG signals were recorded with Maestro Software (9.03 AS, MED-EL, Austria) following established protocols^26^. Potentials were acquired using 2 stimulus polarities, condensation (CON) and rarefaction (RAR). For each polarity, 100 epochs were recorded, and the responses were subtracted to obtain the cochlear microphonic (CM/DIF) signal.

Pure-tone stimulation was initially performed at 250, 500, 1000, and 2000 Hz on electrodes 1, 4, 7, and 10, with electrode 1 positioned at the apical tip of the array. If a clear response was visually detectable at any of these electrodes, broadband (multitone) stimulation was subsequently applied at that site. When participants provided consent, additional recordings were obtained from further electrode positions, typically again including electrodes 1, 4, 7, and 10 and, in some cases, extending beyond these positions. Electrodes were grouped according to cochlear location as follows: apical (electrodes 1 to 3), midpeak (electrodes 4 to 7), and basal (electrodes 8 to 12).

Two multitone stimuli were used, adapted from Polak et al^26^. Multitone 1 comprised 500, 1000, and 2000 Hz, while multitone 2 additionally included 250 Hz. Pure-tone and multitone stimuli were presented at 10 dB hearing level (HL) above each participant’s hearing threshold. For multitone stimulation, the 500 Hz threshold served as the reference for level calibration.

Signal preprocessing was identical to that described by Schuerch et al^27^. For frequency-domain analysis, signals were zero-padded to achieve a frequency resolution (bin width) of 25 Hz, and a Fourier transformation was performed using SciPy’s FFT implementation^28,29^. SNR was calculated using the +/− averaging method^22^.

Two methods were used to analyze ECochG responses elicited by pure-tone and multitone stimuli.

### Expert analysis

Three experts independently assessed the ECochG recordings using the platform Labelbox^30^. The interface displayed the time-domain signals on the left, showing (i) the DIF response and (ii) the CON and RAR responses, and the frequency-domain representations on the right, showing (iii) and (iv) their respective FFT traces. An example of a multitone response is shown in Figure 1. For pure tones, experts determined whether an ECochG response was present (dichotomous decision). For multitone stimuli, they first evaluated whether an overall response was present (dichotomous). If confirmed, raters then assessed which spectral components were present. A response had to be provided for every signal before the next was displayed. Responses were classified as detectable when at least 2 of the 3 experts judged them to be present. Inter-rater agreement was calculated using Fleiss’ Kappa and interpreted according to Landis and Koch^31,32^. To compare signal amplitude distributions across electrodes and frequencies, the electrode with the largest amplitude for each patient–frequency pair was selected from all detectable signals. For multitone stimuli, the electrode corresponding to the highest amplitude at the respective spectral component in the Fourier transform was used instead. Cohen’s d was calculated as proposed by J. Cohen^33^.

**Figure 1:**
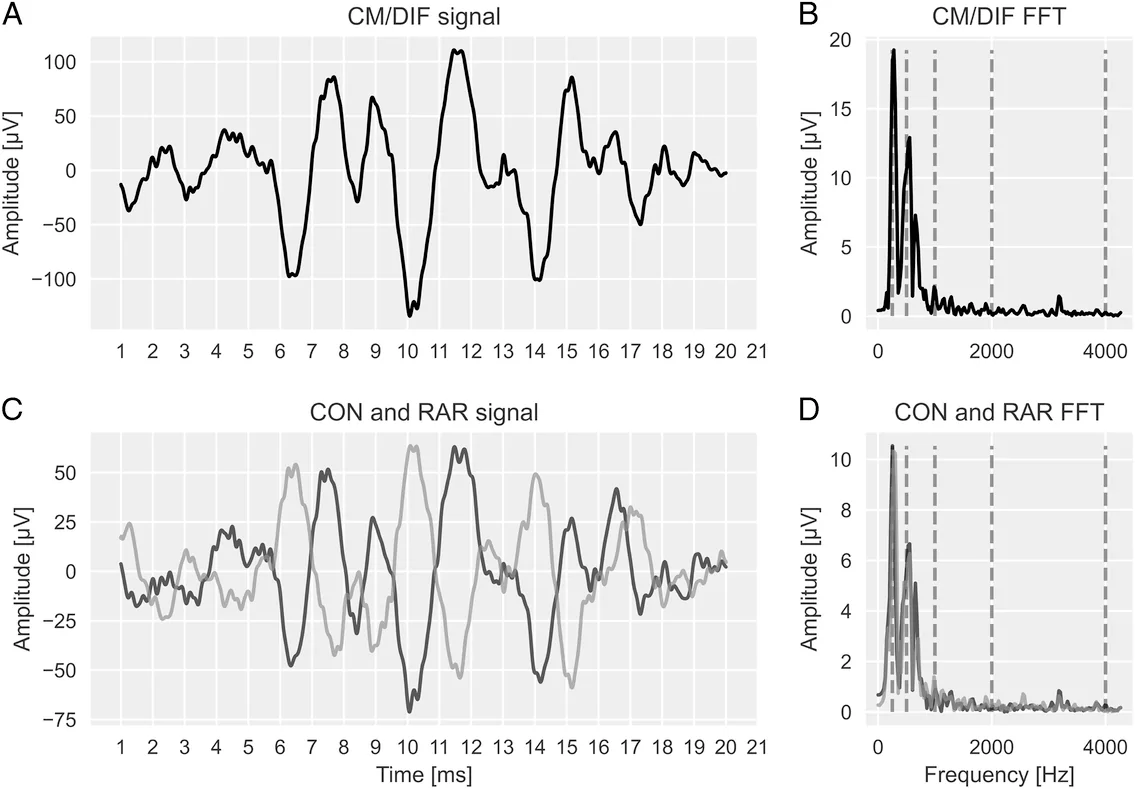
Sample image of a multitone 2 stimulus as presented to the labelers. The CM/DIF signal is shown in subplot (*A*) with the corresponding condensation and rarefaction signals shown in subplot (*C*). The respective Fourier transformations are shown in subplots (*B*) and (*D*). The expected spectral components for the multitone are represented by the vertical gray lines in subplots (*B*) and (*D*).

### Binning method

To objectively assess responses to multitone stimuli, a binning method adapted from previous studies^11,34,35^ was applied. For each spectral component, the amplitude from the bin centered around the respective frequency (target frequency ±12.5 Hz) was selected. The signal’s local standard deviaton (SD) at the amplitude was estimated using 3 adjacent bins on either side of the target bin (±75 Hz total). This SD was regarded as equivalent to the local noise. A response was classified as significant if its amplitude exceeded 2 SDs of the estimated noise, assuming a mean of zero in the absence of a response. Sensitivity and specificity were computed according to the standard formulas described by Altman and Bland, with the experts assessment considered as the ground truth^36^.

## Results

Of the 21 study participants, 12 were female and 9 were male, with a mean age of 59.6 years. The predominant etiologies of hearing loss were unknown progressive hearing loss (n=12), genetic hearing loss (n=4), Menière disease (n=2), and infectious disease (n=2). Electrode arrays were primarily Flex 28 (16 participants), with Flex 24 used in 5 cases. The time of measurement (ToM) postimplantation ranged from 6 to 174 months (mean 42.05 mo). The lfPTA across all participants was 88.1 dB (IQR: 81.2 to 95.0) (Table 1).

**Table 1:** Demographics for all implanted patients

Subject ID	Sex	Age	Side	Course/Etiology	Array	ToM (mo)	lfPTA (dB)
PO_0_	F	60	R	Progressive HL	Flex 28	10	67.5
PO_1_	M	74	R	Progressive HL	Flex 28	17	108.75
PO_2_	M	75	L	Progressive HL	Flex 24	46	63.75
PO_3_	M	80	L	Congenital genetic	Flex 28	9	85.0
PO_4_	F	27	L	Congenital genetic	Flex 28	20	101.25
PO_5_	F	66	R	Progressive HL	Flex 28	28	92.5
PO_6_	F	73	R	Meniere disease	Flex 28	78	90.0
PO_7_	M	82	L	Progressive HL	Flex 28	75	113.75
PO_8_	F	25	R	Congenital genetic	Flex 28	57	85.0
PO_9_	F	44	R	Progressive HL	Flex 28	22	83.75
PO_10_	F	61	R	Progressive HL	Flex 24	13	97.5
PO_11_	F	73	L	Progressive HL	Flex 28	70	98.75
PO_12_	M	50	R	Meningitis	Flex 28	11	80.0
PO_13_	F	68	L	Progressive HL	Flex 28	22	95.0
PO_14_	F	52	R	Congenital genetic	Flex 24	174	93.75
PO_15_	M	50	L	Meningitis	Flex 28	6	75.0
PO_16_	M	67	R	Meniere disease	Flex 28	7	106.25
PO_17_	M	56	R	Sudden HL	Flex 28	11	91.25
PO_18_	M	75	R	Progressive HL	Flex 28	70	95.0
PO_19_	F	63	R	Progressive HL	Flex 24	131	90.0
PO_20_	F	32	L	Progressive HL	Flex 24	6	36.25
Mean	—	59.67	—	—	—	42.05	88.10

HL indicates hearing loss; lfPTA, low frequency pure tone average; ToM, time of measurement.

### Expert analysis

First, all signals were analyzed and rated by 3 independent experts, analogous to the current gold standard of ECochG interpretation. Inter-rater agreement for pure tones and multitone stimuli is presented in Figure 2. Pure tones consistently achieved substantial to almost perfect agreement across frequencies, with the highest values near 0.85 at 500 Hz and 1000 Hz.

**Figure 2:**
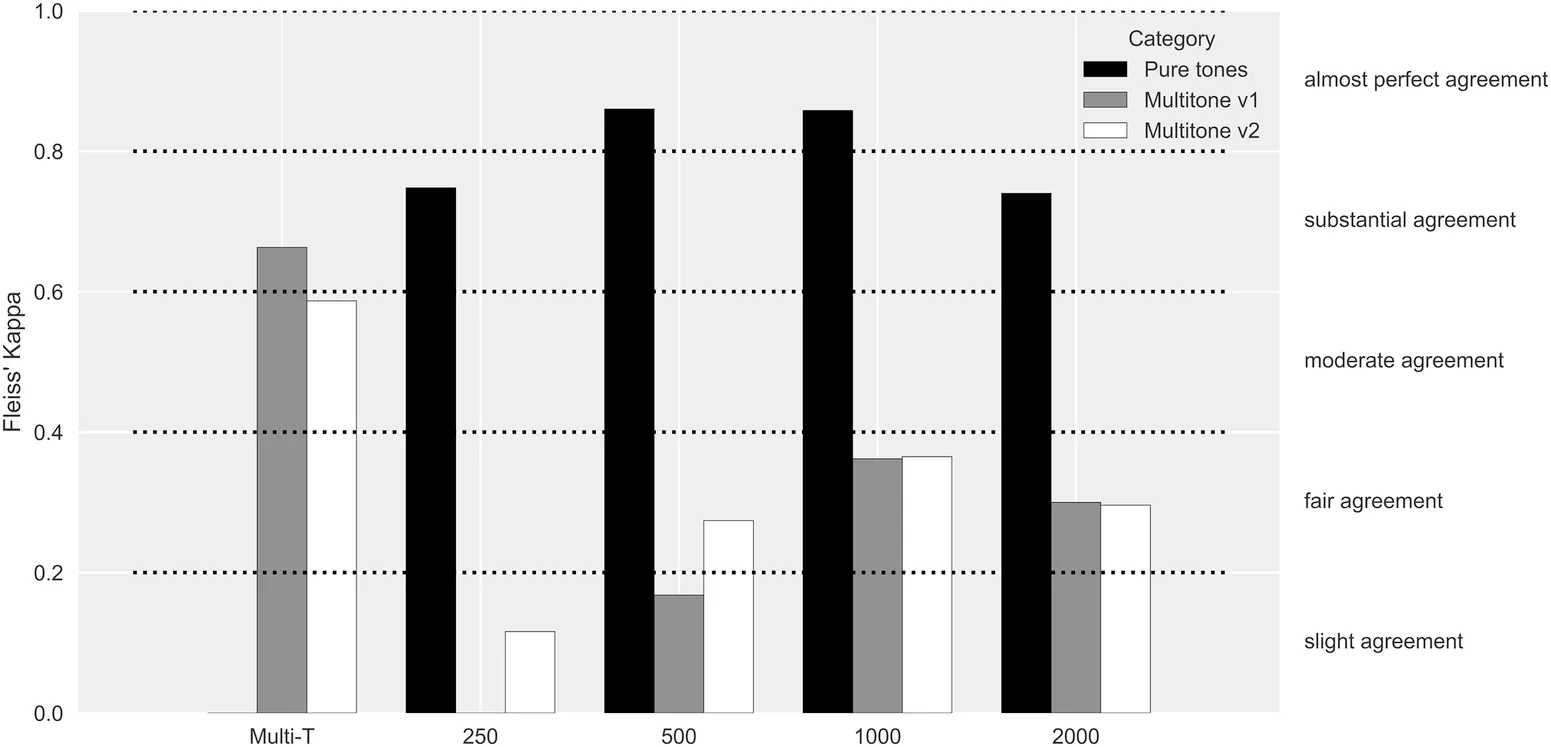
This figure presents the Fleiss’ Kappa values for the overall multitone signal (“Multi-T”) and of the individual spectral components of pure tones (black) and multitones (gray, light gray). The interpretation of Fleiss’ Kappa values, adapted from Landis and Koch, is displayed on the right. Adaptations are themselves works protected by copyright. So in order to publish this adaptation, authorization must be obtained both from the owner of the copyright in the original work and from the owner of copyright in the translation or adaptation.

For multitone stimuli, agreement differed between the overall signal and its spectral components. While overall multitone agreement (i.e., agreement on whether an ECochG response was present) was moderate to substantial for both multitone 1 and multitone 2, values for multitone 1 tended to be slightly higher. In contrast, spectral component agreement (i.e., agreement on whether a specific frequency component such as 500 Hz was present in the multitone signal) was generally lower. A frequency-dependent trend emerged: lower spectral components yielded lower agreement (250 Hz: slight; 500 Hz: fair), and agreement increased at higher spectral components (1000 Hz and 2000 Hz: fair).

For pure-tone response patterns, considering the recordings from different electrode sites, at least one ECochG response was detected at each frequency for every subject. When examining the response patterns of the electrodes with the largest amplitudes for each patient–frequency pair, the median responses were generally located in the mid-peak region with a generally wide IQR: 250 Hz: electrode 7 (IQR: 1.0 to 7.0), 500 Hz: electrode 4 (IQR: 4.0 to 10.0), 1000 Hz: electrode 7 (IQR: 4.0 to 10.0), 2000 Hz: electrode 5.0 (IQR: 1.0 to 7.75).

For multitone stimulation, expert labeling identified 17/21 participants with a detectable overall signal on at least one electrode. At 500 Hz, responses were localized primarily in the mid-peak region (median electrode: 7; IQR: 4.5 to 8.5). As stimulus frequency increased, the distribution of responses shifted progressively toward the basal region, with median electrode positions of 8.5 (IQR: 4.75 to 10.0) at 1000 Hz and 9.5 (IQR: 7.0 to 10.0) at 2000 Hz. Conversely, responses to the 250 Hz stimulus were also situated within the basal region (median electrode: 10; IQR: 7.75 to 10.0). An example of signal change across electrodes and stimulation modalities is shown in Figure 3.

**Figure 3:**
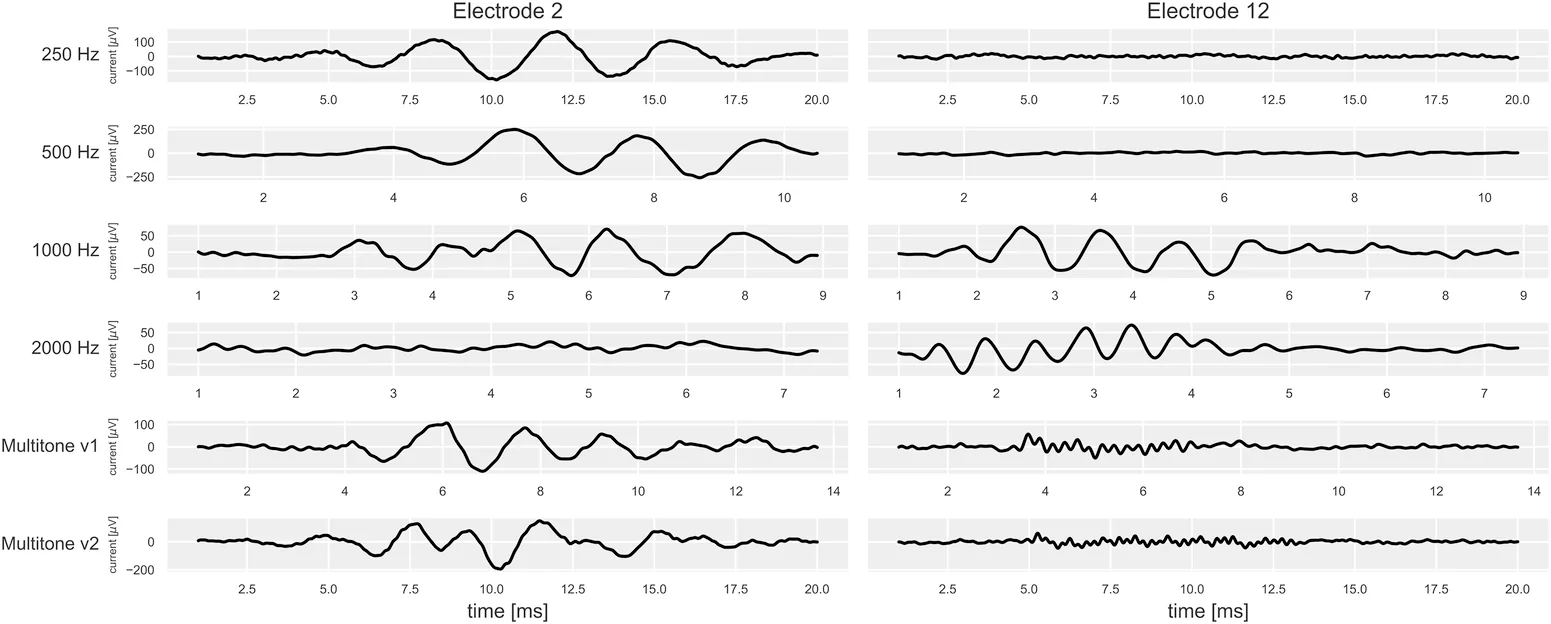
The variation in signal characteristics across different electrodes. The first 4 rows represent pure-tone frequencies, while the bottom 2 rows display multitone v1 and v2, respectively. Signals recorded from electrode 2 are presented on the left column, whereas those from electrode 12 are shown on the right row. Time measured in milliseconds is shown on the *x*-axis while current measured in microvolt is shown on the *y*-axis.

The signal-to-noise ratio (SNR) comparison for pure-tone and multitone stimulation is shown in Figure 4. Across all conditions, SNR values mostly ranged between 1.5 and 4 dB, indicating only a modest dominance of the signal over the background noise and reflecting the difficulty of unequivocal visual classification by experts. Positively identified responses exhibited slightly higher mean SNRs than negative ones, but with substantial overlap between the distributions and a correspondingly small effect size. Pure-tone stimulation at 500 and 1000 Hz yielded the highest SNR values, with 1000 Hz showing the larger effect size of the two. For multitone stimulation, SNRs were comparatively lower; however, the difference between positively and negatively rated responses was still evident in the corresponding effect size (Cohen’s *d=*0.46).

**Figure 4:**
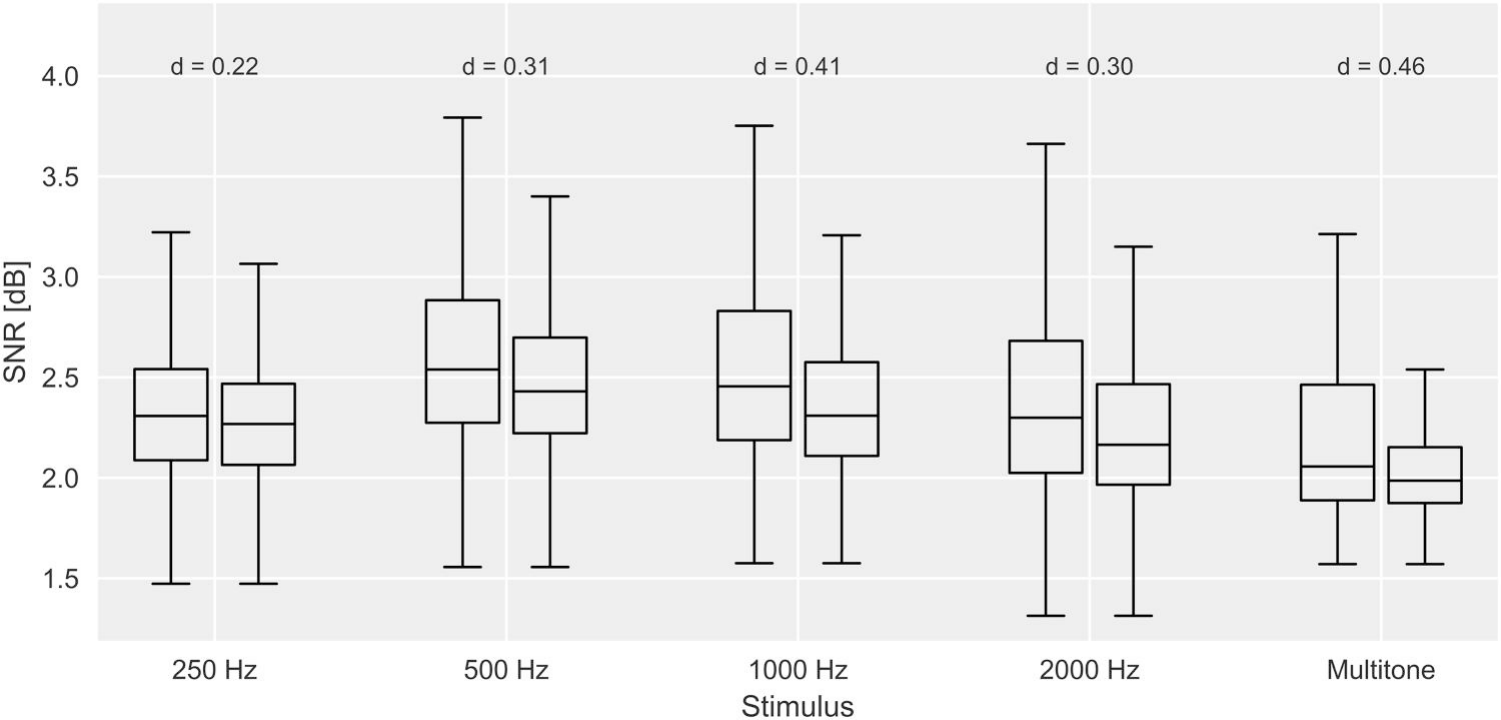
The comparison of the SNR across different conditions is shown in Figure 4. For each group, the left plot shows signals that were rated as positive by the experts, whereas the right plot contains signals that were rated as negative. SNR measured in dB is shown on the *y*-axis, while the analyzed conditions are shown on the *x*-axis. Cohen's d is shown above each condition.

### Binning method

In a second step, we examined whether an objective binning-based method would yield results comparable to expert analysis. Sensitivity and specificity for pure-tone and multitone stimulation were calculated separately for each frequency (Table 2).

**Table 2:** Sensitivity and Specificity for pure tones and multitones across different frequencies

	Pure Tones		Multitones	
Frequency (Hz)	Sensitivity	Specificity	Sensitivity	Specificity
250	0.73	0.54	0.55	0.61
500	0.88	0.38	0.47	0.48
1000	0.92	0.29	0.89	0.38
2000	0.93	0.25	0.84	0.47

For pure tones, sensitivity was generally high across all tested frequencies, with a gradual increase at higher stimulation frequencies (from 0.73 at 250 Hz to 0.93 at 2000 Hz). In contrast, specificity values were clearly lower and showed an opposite frequency dependence, decreasing from 0.54 at 250 Hz to 0.25 at 2000 Hz. This pattern reflects a trade-off between sensitivity and specificity.

For multitone stimuli, a similar overall trend emerged, with increasing sensitivity and decreasing specificity toward higher frequencies, albeit with some deviations at individual frequencies. Sensitivity values were lower than for pure tones (from 0.55 at 250 Hz to 0.89 at 1000 Hz), whereas specificity was higher across all frequencies (from 0.61 at 250 Hz to 0.38 at 1000 Hz).

## Discussion

This study compared the diagnostic value of pure-tone and multitone ECochG stimuli in a stable postoperative setting. Pure-tone stimulation generated near-sinusoidal waveforms that experts identified readily and with high inter-rater agreement, whereas multitone stimulation produced complex, variable responses whose morphology depended on measurement site and residual hearing. Although experts generally detected the presence of an overall multitone response, neither subjective nor objective analyses yielded sufficiently reliable spectral-component-specific information.

### Expert agreement

Inter-rater agreement for pure-tone ECochG was substantially higher than for multitone recordings, as reflected by Fleiss’ Kappa. For pure tones, the observed agreement levels were comparable to previous reports in similar postoperative settings (eg, Schuerch et al^37^), underlining that experts can reliably judge the presence or absence of a response when the stimulus and expected waveform are well defined.

Even for pure tones, it is known that the CM response, although dominated by the stimulus frequency, often deviates from a strict sinusoid because of the superposition with harmonic components, including the so-called auditory nerve neurophonic (ANN)^38^. Nevertheless, experts usually have a clear internal template of what a “typical” pure-tone ECochG waveform should look like at a given frequency and level. In contrast, multitone stimulation produces a superposition of several frequency components (CM and ANN), whose relative contribution varies with residual hearing, cochlear location, and electrode position. In our data, this resulted in considerable variability of waveform morphology across electrodes and participants.

This variability, combined with comparatively low SNR, likely contributed to the reduced agreement for multitone stimuli. While raters still reached moderate to substantial agreement for the overall presence of a multitone response, their confidence evidently diminished when judging individual spectral components, especially at 250 Hz and 500 Hz, where agreement dropped to slight or fair levels. These findings highlight that experts currently lack a robust mental template for multitone ECochG signals, in contrast to the more predictable morphology of pure-tone responses.

### Maximal amplitude patterns

In participants with measurable residual hearing extending into the higher frequency range, ECochG amplitudes followed a plausible tonotopic pattern, with lower frequencies recorded more apically and higher frequencies more basally. Similar tonotopic distributions of ECochG maxima have been described previously for pure-tone and multifrequency stimulation^17,25^.

However, many CI candidates typically present with a left-corner audiogram, characterized by measurable thresholds predominantly at low frequencies and absent or very poor hearing at higher frequencies. This pattern was also reflected in our cohort. When examining the electrodes with the highest ECochG amplitudes, we frequently observed a basal shift of the maxima, even for relatively low stimulation frequencies. This observation is consistent with postoperative ECochG reports^37,39^, suggesting that the distribution of residual function along the cochlea may be substantially altered compared with the prepathological tonotopic map.

Several mechanisms may contribute to this basal shift. First, damage to outer hair cells in the basal turn is common in CI candidates and may distort or displace the region of maximal mechanical and electrophysiological response^40^. Second, the presence of the electrode array itself can modify cochlear mechanics and local fluid dynamics, thereby altering the effective place of maximal stimulation^41^. Third, a lateral wall position of the electrode has been found to produce a more basal pattern compared with a perimodiolar position^42,43^. Fourth, relatively high stimulation levels may induce the spread of excitation and partial loss of strict tonotopic specificity^44^. Although we aimed to keep stimulation moderate by setting levels at 10 dB HL above behavioral thresholds, the absolute levels required in ears with severe hearing loss can still be high.

Arrays of different lengths were used in this study. However, we consider it unlikely that electrode length substantially contributed to the observed basal shift. The values for tonotopic positions of Flex24 electrodes were in the range of Flex28 electrodes and the most extreme values were observed in the Flex28 group exclusively. Moreover, the basal shift was also present for the 250 Hz stimulus and neither array extended to this tonotopic region. Thus, maximum amplitudes would be expected at electrode 1 for both array types. They were, however, observed more basally in either group.

Overall, the observed patterns of maximal ECochG amplitude likely reflect a combination of residual cochlear pathology, electrode-induced mechanical alterations, and level-dependent spread of excitation.

### Objective analysis

Overall, both pure-tone and multitone responses were characterized by relatively low SNRs, typically between 1.5 and 4 dB, with only modest differences between positive and negative expert ratings. This resulted in small effect sizes for most conditions and illustrates why experts sometimes disagreed on whether a given trace contained a genuine ECochG response. Interestingly, effect sizes for multitone stimuli were slightly higher than those of pure tones, despite the lower absolute SNR values, indicating that multitone responses still carried discriminative information, but in a more complex spectral pattern.

The binning-based objective method used in this study was chosen because it is comparatively simple to implement and conceptually aligned with approaches proposed by other groups^10,11,45^. It has also been applied to multitone data in an intraoperative context to detect ECochG amplitude drops^18^. However, previous work typically used binning or related procedures without direct comparison to expert ratings, limiting conclusions about their diagnostic robustness.

In our data set, the binning method showed high sensitivity but low specificity for pure tones, particularly at higher frequencies, suggesting that true responses were detected reliably, but at the cost of numerous false positives. For multitone stimuli, sensitivity was generally lower at 250 Hz but approached pure-tone levels at higher frequencies, while specificity tended to be similar to or higher than that of pure tones at the lower frequencies. Thus, the method did not simply fail for multitone stimuli but exhibited a frequency-dependent trade-off between sensitivity and specificity.

Two factors likely contributed to these limitations. First, the binning algorithm assumed a zero-mean noise floor. In practice, residual noise remained after preprocessing and stitching artifacts produced spurious spectral peaks that were occasionally classified as responses, reducing specificity. The small SNR differences between positive and negative traces reinforce that noise remains an important confound even in processed signals. Second, slight detuning between the FFT peak and the nominal stimulation frequency was not uncommon. Human raters tolerated such deviations and could discount isolated noise-like peaks, whereas the fixed-bin algorithm was less flexible, which reduced sensitivity in some cases. Together, these findings suggest that simple amplitude-based binning is insufficient for robust classification, particularly for complex multitone responses.

### Implications for intraoperative monitoring with multitones

In the intraoperative setting, a key advantage of multitonal over pure tone stimulation is the theoretical ability to detect amplitude drops associated with passing the characteristic frequency, making intraoperative application the ultimate goal. However, a solid electrophysiological understanding of the recorded responses must first be established. In this study, we demonstrated that overall signal responses could be identified consistently. In contrast, the spectral components showed high variability and limited reproducibility, resulting in unreliable spectral response identification. These limitations should be addressed before the intraoperative application appears viable.

### Strengths, limitations, and future directions

This study provided a systematic evaluation of postoperative multitone ECochG using a standardized stimulation and recording protocol and 3 independent, experienced raters. The combined use of expert ratings and an objective binning method allowed a direct comparison of subjective and algorithmic classification performance for both pure-tone and multitone stimuli.

Several limitations must be acknowledged. The sample size was modest, which restricted subgroup analyses and may limit generalizability to specific etiologies or array types. Measurement time constraints meant that recordings could not be obtained from every electrode in all participants, so spatial sampling along the array remained incomplete. These factors may have reduced the ability to fully characterize multitone response patterns across the entire cochlea. In addition, in patients with especially pronounced high-frequency hearing loss, certain spectral components may be absent altogether, limiting the utility of multitone stimulation in this population. Finally, we used a relatively simple objective method. While the binning approach is easy to implement and clinically feasible, more advanced methods have already shown superior performance for pure-tone ECochG^46^, including machine-learning approaches that nearly match expert performance^22,27^.

Future work should focus on improving the reliable detection of true responses, both visually and algorithmically. For visual analysis, further noise reduction through longer recording durations, optimized averaging, improved artifact rejection, and refined filtering may enhance clarity and confidence. For objective analysis, algorithms that can tolerate slight frequency detuning and incorporate local spectral context (for example, adaptive peak-tracking rather than fixed bins, or matched-filter strategies) appear promising. Finally, machine-learning models trained on large, expertly labeled data sets could capture the multivariate structure of multitone responses more effectively.

## Conclusion

In a stable postoperative setting, multitone ECochG was feasible and produced detectable signals. However, response characteristics varied with electrode position and residual hearing, yielding more complex patterns than pure tones. This complexity hindered reliable identification of individual spectral components by experts and by objective methods. Future work might enhance signal quality through noise reduction and longer recordings, and develop more sophisticated algorithms for robust spectral component detection and interpretation.
